# Zerumbone suppresses the motility and tumorigenecity of triple negative breast cancer cells via the inhibition of TGF-β1 signaling pathway

**DOI:** 10.18632/oncotarget.6441

**Published:** 2015-11-30

**Authors:** Sangmin Kim, Jeongmin Lee, Myeongjin Jeon, Jeong Eon Lee, Seok Jin Nam

**Affiliations:** ^1^ Department of Surgery, Samsung Medical Center, Sungkyunkwan University School of Medicine, Gangnam-gu, Seoul, South Korea; ^2^ Department of Health Sciences and Technology, Samsung Advanced Institute for Health Sciences and Technology, Gangnam-gu, Seoul, South Korea

**Keywords:** zerumbone, TGF-β1, Smad3, cell invasion, triple negative breast cancer

## Abstract

Aberrant transforming growth factor-β (TGF-β) plays an important role in the development of cancer such as tumor metastasis and invasion. TGF-β-responsive gene signature is highly activated in chemotherapy-treated triple negative breast cancer (TNBC). Here, we investigated the effect of zerumbone (ZER) on TGF-β1 signaling pathway and tumorigenecity of TNBC cells. Our results showed that the level of TGF-β1 mRNA expression and cell invasiveness were higher in TNBC cells than in non-TNBC cells. On the other hand, the cell motility of TNBC cells was completely suppressed by LY2109761, a novel selective TGF-β receptor type I/II (TβRI/II) dual inhibitor. In addition, FN and MMP-2 expression, which play an important role on cell motility in various cancer cells, were dose-dependently decreased by LY2109761. TGF-β1 increased FN, MMP-2 and MMP-9 expression in HCC1806 TNBC cells. TGF-β1-induced MMP-9 expression was decreased by both a MEK inhibitor, UO126, and a smad3 inhibitor, SIS3. Induction of FN and MMP-2 by TGF-β1 was just decreased by SIS3. Overexpression of smad3 significantly increased FN, MMP-2, and MMP-9 expression. Interestingly, ZER significantly suppressed TGF-β1-induced FN, MMP-2, and MMP-9 expression in HCC1806 cells. In addition, ZER completely decreased TGF-β1-induced the phosphorylation of smad3. Finally, we observed that ZER suppressed the tumorigenecity such as tumor volume, weight, Ki67 expression, and metastasis in TNBC cells xenograft models. Taken together, we demonstrated that ZER suppresses TGF-β1-induced FN, MMP-2, and MMP-9 expression through the inactivation of smad3 and inhibits the tumorigenecity of TNBC cells. Therefore, we suggest that ZER may act as a promising drug for treatment of TNBC.

## INTRODUCTION

Triple-negative breast cancer (TNBC) accounts for 15% to 20% of breast cancers and is a heterogeneous disease [1, 2]. TNBC are defined by their failure to express the estrogen receptor (ER), progesterone receptor (PR), and human-epidermal growth factor receptor-2 (HER2) proteins [3]. Of all subtypes of breast cancer, TNBC tends to occur in younger age with pre-menopausal women and occurs more frequently in African American women [4, 5]. In addition, TNBC is typically associated with poor prognosis when compared with other breast cancer subtypes [4-7]. Especially, highly recurrent rate of TNBC is associated with aggressive phenotype, chemotherapy resistance, and the lack of clinically therapeutic targets [6, 8]. To date, no therapeutic agents have been clinically approved for treatment of TNBC. Thus, many researchers focus on the development of new therapeutic agents for treatment of TNBC.

Transforming growth factor-βs (TGF-β) are multifunctional growth factors that play particularly complex roles in the growth, progression, and metastatic potential of cancers [9]. TGF-β1 is highly expressed in a various cancers such as TNBC, prostate, and lung cancer and leads to tumor promotion and metastasis [10, 11]. In late-stage human tumors, the level of TGF-β1 expression frequently increases and aberrant TGF-β1 expression is associated with more aggressive tumors and poor prognosis [10, 12]. One of several targeted strategies for the treatment of patients with TNBC is the neutralization of TGF-β [13]. *Nam et al*. reported that treatment of mice with a monoclonal anti-TGF-β antibody strongly suppresses development of either lung- or bone metastases in 4T1 syngeneic mammary tumor models [13]. In addition, induction of epithelial-mesenchymal transition (EMT) by TGF-β facilitates the invasiveness of breast cancer cells [14-16].

FN is a large adhesive glycoprotein and plays an important role on cell migration, invasion, and differentiation [17]. FN augments human carcinoma cell growth through upregulation of inflammatory factors such as COX-2 and IL-8 in lung cancer cells [18, 19]. In addition, FN triggers MMP-2 and MMP-9 expression which are involved with cell invasion and migration through binding of RGD sequence of α5β1 integrin in several human carcinoma cells, including breast cancer cells [18, 19]. Aberrant MMP-2 and MMP-9 expression is apparent in breast cancers and these proteins release often associated with tumor invasion and metastasis [20].

Zerumbone (ZER) was first isolated from a Southeast Asian ginger sesquiterpene and has a wide range of pharmacologic effects, such as anti-inflammation, antioxidant agent, and anti-carcinogenic agents in a variety of tumor cells including breast, colon, and gastric cancer [21-23]. We also reported that ZER suppresses IL-1β-induced cell invasion by inhibiting IL-8 expression and MMP-3 expression in TNBC cells [24]. In addition, ZER completely reduces angiogenesis through the inhibition of NF-κB activity and NF-κB-dependent proangiogenic genes such as VEGF and IL-8 [25, 26]. ZER inhibits cell growth of various cancer cells through the induction of apoptosis *via* the down-regulation of surviving and Bcl2 [21]. *Abdelwahab et al.* has suggested that ZER is a promising chemotherapeutic agent through the cell cycle of G2/M phase and the suppression of IL-6 secretion in cervical and ovarian cancer cells [27].

In this study, we evaluated the inhibitory effect of ZER on TGF-β1-induced FN, MMP-2, and MMP-9 expression in TNBC cells. We found that the level of TGF-β1 expression was higher in TNBC than in non-TNBC. The activation of smad3 by TGF-β1 was closely associated with the induction of FN, MMP-2, and MMP-9 expression. In contrast, blocking of smad3 significantly declined TGF-β1-induced FN, MMP-2, and MMP-9 expression. We found for the first time that ZER completely abolished TGF-β1-induced smad3 phosphorylation and then, reduced TGF-β1-induced FN, MMP-2, and MMP-9 expression as well as the tumorigenecity of TNBC cells.

## RESULTS

### The level of TGF-β1 expression and cell invasion is higher in TNBC than in non-TNBC

**Figure 1:**
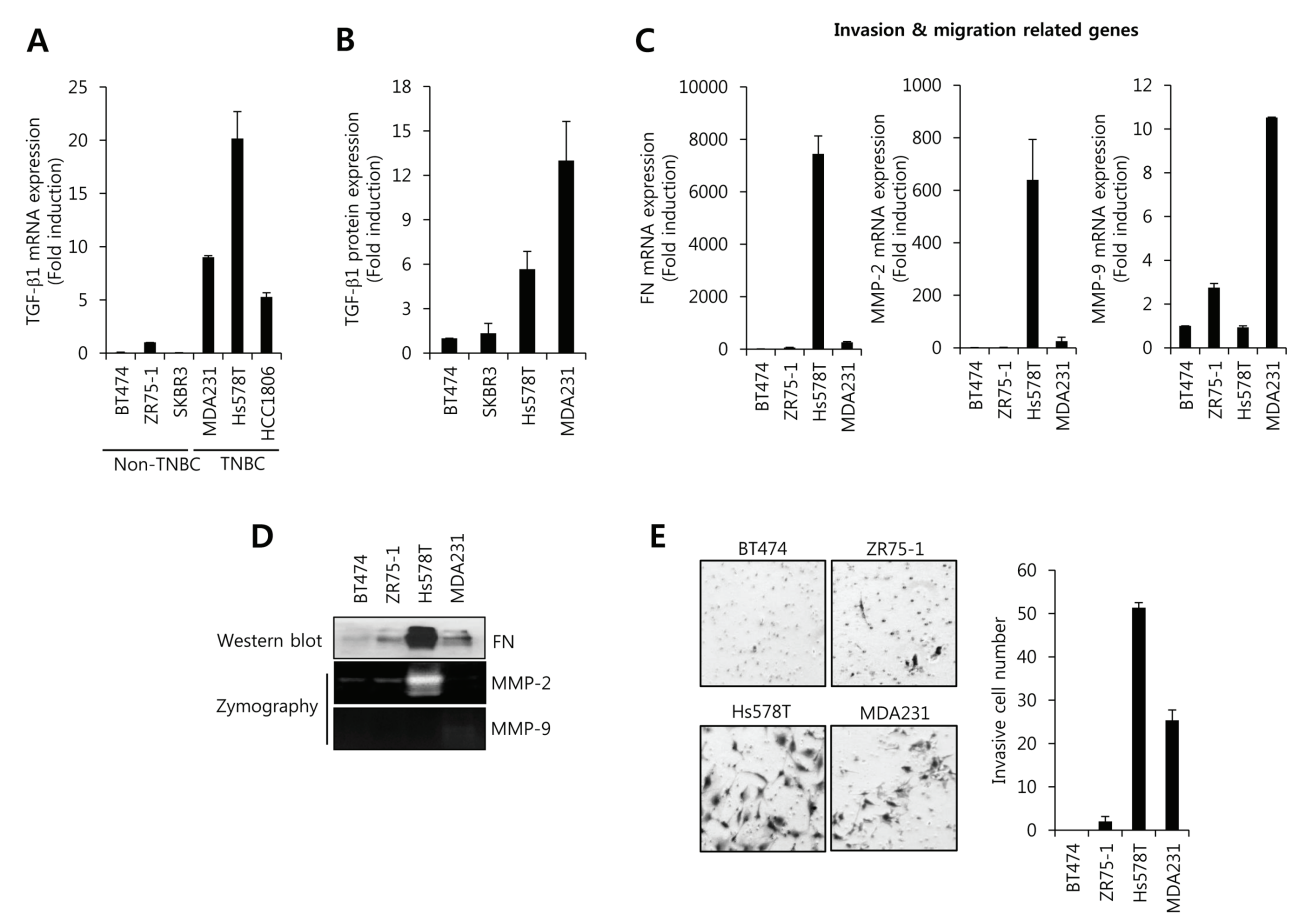
The level of TGF-β1 expression and cell invasion is higher in TNBC cells than in non-TNBC cells After serum-starvation for 24 h, breast cancer cells were harvested for detection the levels of TGF-β1 mRNA **A.** and protein **B.** expression using the real-time PCR and ELISA, respectively. **C.** The levels of FN, MMP-2, and MMP-9 mRNA expression were analyzed by real-time PCR in breast cancer cells. **D.** The levels of FN, MMP-2, and MMP-9 protein expression were analyzed by western blotting and zymography, respectively. **E.** After seeding of 5×105 cells/well, breast cancer cells further incubated for 48 h. After 48 h incubation, cells on the bottom side of filter were fixed and stained. Cell morphology was analyzed using a CK40 inverted microscope. The results are representative of three independent experiments. Con: control.

Elevated TGF-β1 is correlated with a high incidence of distant metastasis of various tumor cells and promotes epithelial to mesenchymal transition (EMT), ECM degradation, cell migration, cell invasion, and angiogenesis [11, 28]. Thus, we investigated the level of TGF-β1 mRNA expression between in non-TNBC cells and in TNBC cells. Interestingly, our results showed that TGF-β1 mRNA and protein expression was significantly increased in TNBC cells compared with non-TNBC cells (Figure 1A and 1B). The level of TGF-β1 mRNA expression in MDA231 and Hs578T cells was significantly increased by 9.0-fold and 20.2-fold of the level of ZR75-1 cells, respectively (Figure 1A). In addition, the levels of FN and MMP-2 mRNA expression were also increased in TNBC cells, although MMP-9 expression did not show a sharp difference (Figure 1C). Especially, the levels of FN and MMP-2 protein expression were significantly increased in Hs578T cells (Figure 1D). Furthermore, we observed that the invasion capacity of TNBC cells also was far superior to non-TNBC (Figure 1E). Therefore, we demonstrated that the increasing amount of TGF-β1 may be correlated with the invasion and migration of TNBC cells.

### The migration and invasion of TNBC cells is suppressed by LY2109761 treatment

**Figure 2:**
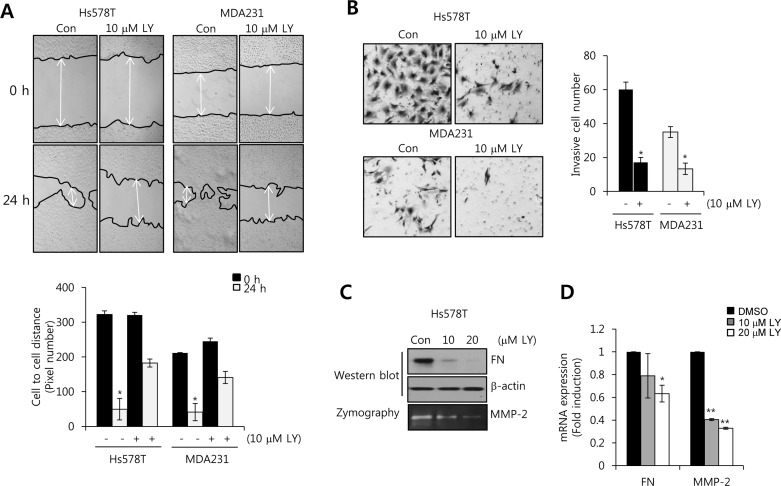
The transforming growth factor (TGF)-β receptor I/II inhibitor LY2109761 decreases FN and MMP-2 expression in TNBC cells **A.** After serum starvation for 24 h, Hs578T and MDA231 cells were treated with or without 10 μM LY2109761 for 24 h. The migrating ability of TNBC cells was analyzed using the wound healing assay. **B.** After seeding 5 × 105 cells/well, TNBC cells were treated with or without 10 μM LY2109761 for 24 h in serum-containing media. Invasive cells were analyzed with the cell invasion assay (Boyden chamber assay). **C.** Under the same conditions, FN and MMP-2 protein expression levels were analyzed by western blotting and zymography, respectively. **D.** Under the same conditions, FN and MMP-2 mRNA expression levels were analyzed by real-time PCR. Results are representative of three independent experiments. Values are means ± standard errors. * *P* < 0.05, ** *P* < 0.01 *vs*. control. Con, control.

To verify the co-relation between TGF-β and motility of TNBC cells, we treated with a dual TGF-β receptor I/II inhibitor, LY2109761, for 24 h in Hs578T and MDA231 cells. As expected, our results showed that the migration of TNBC cells was significantly decreased by LY2109761 in both Hs578T and MDA231 cells (Figure 2A). In addition, invasion capacity of TNBC cells was also suppressed by LY2109761 treatment (Figure 2B). In previous studies, highly expressed FN, MMP-2, and MMP-9 trigger cell invasion and migration in several human carcinoma cells, including breast cancer cells [18, 19]. So, we investigated the level of FN, MMP-2, and MMP-9 expression by LY2109761 in Hs578T cells. Our result showed that the levels of FN and MMP-2 protein expression were decreased by LY2109761 in a dose-dependent manner (Figure 2C). Here, we cannot detect endogenous MMP-9 expression of Hs578T cells (data not shown). The levels of FN and MMP-2 mRNA were decreased to 0.63 ± 0.07-fold and 0.33 ± 0.01-fold of the control level, respectively, at 20 μM LY2109761 treatment (Figure 2D). Furthermore, we investigated the effect of TGF-β receptor I (TGFBRI) and TGF-β receptor II (TGFBRII) siRNA on FN, MMP-2, and MMP-9 mRNA expression. As shown in supplement Figure 1, the levels of FN, MMP-2, and MMP-9 mRNA expression were decreased by TGFBRI or TGFBRII siRNA overexpression, respectively, in a cell-specifically. Therefore, we demonstrated that elevated TGF-β expression may increase the cell invasion and migration through the induction of FN, MMP-2, and MMP-9 in TNBC cells.

### FN, MMP-2, and MMP-9 expression is increased by TGF-β1 treatment in a dose- and time-dependent manner in HCC1806 breast cancer cells

**Figure 3:**
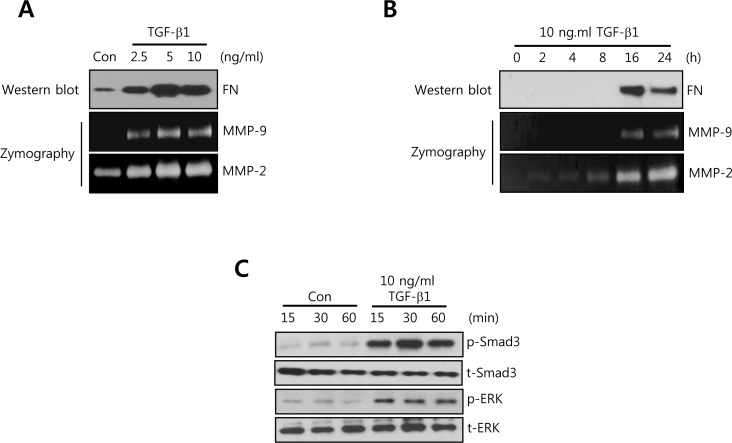
FN, MMP-2, and MMP-9 expression is increased by TGF-β1 treatment in a time- and dose-dependent manner in HCC1806 breast cancer cells **A.** After serum-starvation for 24 h, HCC1806 cells were treated with TGF-1 for the indicated concentrations. **B.** After serum-starvation for 24 h, HCC1806 cells were treated with 10 ng/ml TGF-β1 for the indicated times. The levels of FN protein expression were analyzed by western blotting. The levels of MMP-2 and MMP-9 protein expression were analyzed by zymography using the cell culture media. **C.** After serum-starvation for 24 h, HCC1806 cells were treated with 10 ng/ml TGF-β1 for the indicated times. The levels of smad3 and ERK protein expression were analyzed by western blotting. The results are representative of three independent experiments. Con: control.

To investigate the regulatory mechanism of cell invasion and migration by TGF-β1 in TNBC cells, we chosen the HCC1806 triple negative breast cancer cells. As shown in Figure 1A, the level of TGF-β1 mRNA expression was relatively lower than other TNBC cells. In addition, SUM159 and HCC1806 cells have been known very high TGF-β sensitivity [29]. Thus, we treated HCC1806 cells with TGF-β1 for the indicated dose and time. The levels of FN, MMP-9, and MMP-2 protein expression were dose- and time-dependently increased by TGF-β1 (Figure 3A and 3B). In addition, we observed that the activity of smad3 and ERK was significantly increased by TGF-β1 (Figure 3C). Based on these results, we demonstrated that TGF-β1 directly augments the levels of FN, MMP-2, and MMP-9 through the activation of various signaling molecules in TNBC cells.

### TGF-β1-induced FN, MMP-2, and MMP-9 expression is decreased by smad3-dependent pathway

**Figure 4:**
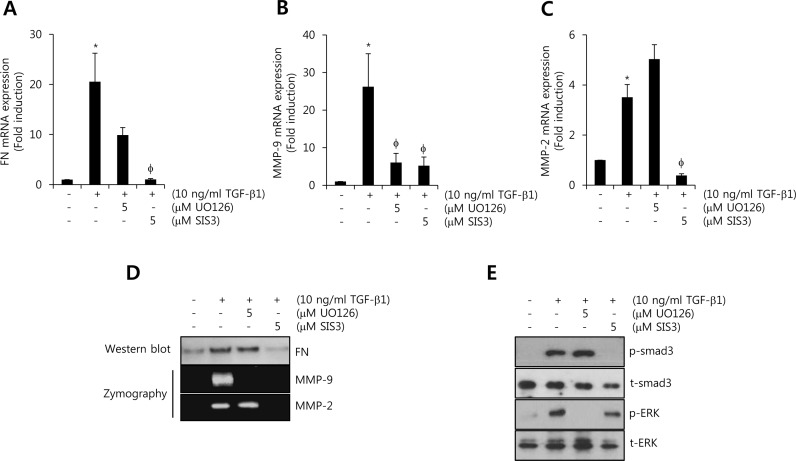
TGF-β1-induced FN, MMP-2, and MMP-9 expression is decreased by smad3-dependent pathway **A.**, **B.**, **C.** After serum-starvation for 24 h, HCC1806 cells were pretreated with 5 μM UO126 and SIS3, respectively, for 30 min, and then they were treated with 10 ng/ml TGF-β1 for 24 h. The levels of FN **A.**, MMP-2 **B.**, and MMP-9 **C.** mRNA were analyzed by real-time PCR. **D.** Under the same condition, the levels of FN protein expression were analyzed by western blotting. The levels of MMP-2 and MMP-9 protein expression were analyzed by zymography using the cell culture media. **E.** After serum-starvation for 24 h, HCC1806 cells were pretreated with 5 μM UO126 and SIS3, respectively, for 30 min, and then they were treated with 10 ng/ml TGF-β1 for 30 min. The levels of smad3 and ERK protein expression were analyzed by western blotting. The results are representative of three independent experiments. Values are means ± standard errors. * *P* < 0.05 *vs*. control. φ *P* < 0.05 *vs*. TGF-β1-treated cells. Con: control.

To investigate the regulatory mechanism of TGF-β1-induced FN, MMP-2, and MMP-9 expressions, we treated HCC1806 cells with 10 ng/ml TGF-β1 for 24 h after pretreatment with UO126 (a MEK inhibitor) and SIS3 (a smad3 inhibitor) for 30 min, respectively. To assess the levels of FN, MMP-2, and MMP-9 mRNA and protein expression, we harvested the cell lysates and culture media, respectively. As shown in Figure 4A, TGF-β1-induced FN mRNA expression was decreased by SIS3 (1.4 ± 0.05-fold of the control level). Induction of MMP-9 mRNA by TGF-β1 also significantly decreased by UO126 (11.5 ± 1.8-fold of the control level) and SIS3 (9.2 ± 3.2-fold of the control level), respectively (Figure 4B). In addition, TGF-β1-induced MMP-2 mRNA expression was decreased by SIS3 (0.24 ± 0.02-fold of the control level) but not by UO126 (Figure 4C). Under the same conditions, our results showed that TGF-β1-induced FN and MMP-2 protein expressions were markedly decreased by SIS3 treatment (Figure 4D). However, TGF-β1/ERK signaling pathway was not affected on TGF-β1-induced FN and MMP-2 protein expressions (Figure 4D). TGF-β1-induced MMP-9 protein expressions were decreased by UO126 and SIS3 treatment, respectively (Figure 4D). Here, we confirmed the effect of each inhibitor on TGF-β1-induced the phosphorylation of ERK1/2 and smad3. As expected, TGF-β1-induced the phosphorylation of ERK1/2 and smad3 was well abolished by UO126 and SIS3, respectively (Figure 4E). Based on these results, we demonstrated that TGF-β1-induced FN, MMP-2, and MMP-9 expressions were regulated through the smad3-dependent pathway in TNBC cells.

### The effect of ZER on cell viability, the level of FN, MMP-2, and MMP-9 mRNA and cell migration

**Figure 5:**
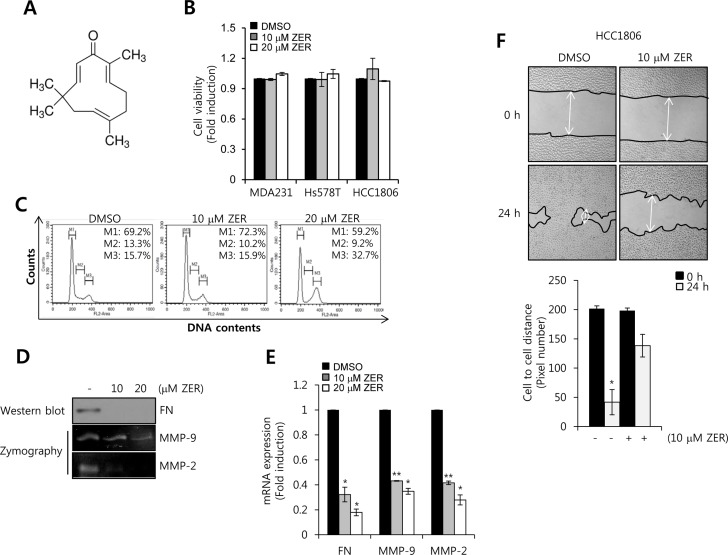
The effect of ZER on cell viability, FN, MMP-2, MMP-9 mRNA expression **A.** The chemical structure of ZER. **B.** After serum starvation for 24 h, HCC1806 cells were treated with ZER at the indicated concentration for 24 h. The viabilities of the cells were analyzed by cell proliferation assay, as described in Materials and Methods. **C.** After serum starvation for 24 h, HCC1806 cells were treated with or without 10 and 20 μM ZER for 24 h, respectively. Cell cycle of HCC1806 cells was analyzed by flow-assisted cell sorting analysis. **D.** Under the same condition, the levels of FN protein expression were analyzed by western blotting. The levels of MMP-2 and MMP-9 protein expression were analyzed by zymography using the cell culture media. **E.** The levels of FN, MMP-2, and MMP-9 mRNA were analyzed by real-time PCR. **F.** After serum starvation for 24 h, HCC1806 cells were treated with or without 10 and 20 μM ZER for 24 h. The migrating ability of HCC1806 cells was analyzed using the wound healing assay. These results are representative of three independent experiments. Values are means ± standard errors. * *P* < 0.05, ** *P* < 0.01 *vs*. DMSO treated cells. Con: control.

To verify the effect of ZER on cell viability, FN, MMP-2, and MMP-9 expressions, we treated HCC1806 cells with 10 and 20 μM ZER for 24 h, respectively. The chemical structure of ZER was indicated in Figure 5A. Our results showed that cell viability had no significant change by 10 and 20 μM ZER treatment in TNBC (Figure 5B). However, we observed that ZER triggered the cell cycle arrest at G2/M phase in HCC1806 cells (Figure 5C) whereas, cell cycle of MDA-MB231 cells did not affect by ZER (data not shown). In previous study, *Xian et al.* reported that ZER was sensitive to NB4 and U937 leukemic cells but resistant to MOLT4 and KT-1 leukemic cells [30]. Therefore, we also suggested that HCC1806 cells were more sensitive to ZER than MDA-MB231 cells.

To investigate the effect of ZER on the basal level of FN, MMP-2, and MMP-9 mRNA expressions in HCC1806 cells, we treated with 10 and 20 μM ZER, respectively, for 24 h. Our results showed that ZER decreased the levels of FN, MMP-2, and MMP-9 protein and mRNA expression in a dose-dependent manner (Figure 5D and 5E). The basal levels of FN, MMP-2, and MMP-9 mRNA expression were reduced to 0.18 ± 0.03-fold, 0.28± 0.04-fold, and 0.35 0.02-fold of the control level at 20 μM ZER treatment (Figure 5E). In addition, we examined the effect of ZER on cell migration of HCC1806. As expected, we observed that the migration rate of HCC1806 cells was significantly decreased by ZER (Figure 5F). Therefore, we suggested that ZER suppressed migration of TNBC cells through the down-regulation of FN, MMP-2, and MMP-9.

### ZER treatment suppresses TGF-β1-induced FN, MMP-2, and MMP-9 expression through the inhibition of smad3 activity

**Figure 6:**
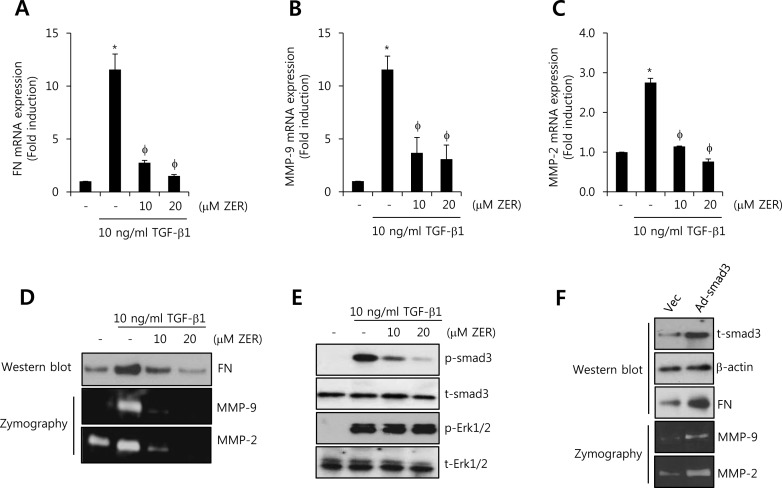
ZER suppresses TGF-β1-induced FN, MMP-2, and MMP-9 expression through the inhibition of smad3 activity After serum-starvation for 24 h, HCC1806 cells were pretreated with 10 and 20 μM ZER, respectively, for 16 h and then they were treated with 10 ng/ml TGF-β1 for 24 h. The levels of FN **A.**, MMP-2 **B.**, and MMP-9 **C.** mRNA were analyzed by real-time PCR. **D.** Under the same condition, the levels of FN protein expression were analyzed by western blotting. The levels of MMP-2 and MMP-9 protein expression were analyzed by zymography using the cell culture media. **E.** After serum-starvation for 24 h, HCC1806 cells were pretreated with 10 and 20 μM ZER, respectively, for 16 h and then they were treated with 10 ng/ml TGF-β1 for 30 min. **F.** After adenoviral smad3 transfection for 24 h, the cells were further incubated in serum-free media for 24 h. The expression of smad3 and ERK was analyzed by western blotting using the cell lysates. The results are representative of three independent experiments. Values are means ± standard errors. * *P* < 0.05 *vs*. control. φ *P* < 0.05 *vs*. TGF-β1-treated cells. Con: control.

Next, we investigated whether ZER regulates TGF-β1-induced FN, MMP-2, and MMP-9 expressions. After pretreatment with 10 and 20 M ZER for 16 h, HCC1806 cells with 10 ng/ml TGF-β1 for 24 h. We observed that FN mRNA expression by TGF-β1 was increased by 11.6 ± 1.5-fold of the control level (Figure 6A). In contrast, TGF-β1-induced FN mRNA expression was suppressed to 1.5 ± 0.1-fold of the control level at 20 μM ZER treatment (Figure 6A). Furthermore, TGF-β1-induced MMP-9 and MMP-2 mRNA expression was completely decreased to 3.1 ± 1.3-fold and 0.76 ± 0.07-fold of the control level at 20 μM ZER treatment, respectively (Figure 6B and 6C). As expected, our results also showed that TGF-β1-induced FN, MMP-2, and MMP-9 protein expressions were abolished by ZER treatment in a dose-dependent manner (Figure 6D). Therefore, we demonstrated that ZER downregulates the levels of FN, MMP-2, and MMP-9 expression through the inhibition of TGF-β signaling pathway in TNBC cells.

Next, we studied the effect of ZER on TGF-β1-induced the phosphorylation of smad3 in HCC1806 breast cancer cells. After pretreatment with 10 and 20 μM ZER for 16 h, the cells were treated with 10 ng/ml TGF-β1 for 30 min. Here, we observed that TGFβ1-induced the phosphorylation of smad3 was completely suppressed by 20 μM ZER treatment (Figure 6E). However, TGFβ1-induced the phosphorylation of ERK1/2 did not affect by ZER treatment (Figure 6E).

To verify the alteration of FN, MMP-9, and MMP-2 expression by smad3 overexpression, HCC1806 cells were transfected with adenoviral vec (Ad-vec) and smad3 (Ad-smad3), respectively, for 48 h. As shown in Figure 6F, the levels of FN, MMP-9, and MMP-2 expression were significantly increased by smad3 overexpression. Therefore, we demonstrated that ZER may suppress TGF-β1-induced FN, MMP-2, and MMP-9 expression through the inhibition of smad3-dependent pathway in TNBC cells.

### ZER suppresses the growth and metastasis of TNBC xenograft tumors

**Figure 7:**
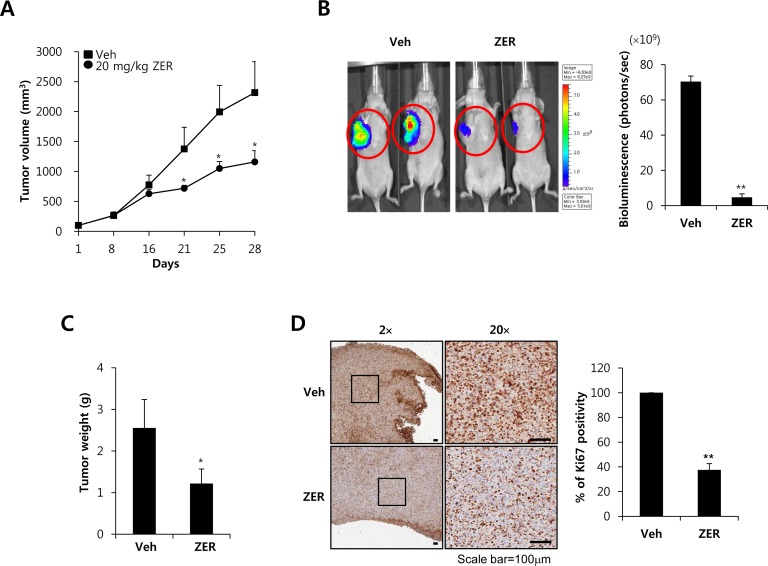
ZER suppresses the growth of MDA-MB231 xenograft tumors **A.** Mice were injected with MDA-MB231 TNBC cells. Tumor sizes from each group (*n* = 5) were analyzed for 28 days. * *P* < 0.05 *vs*. vehicle. **B.** Bioluminescent imaging analysis was analyzed by in a Xenogen IVIS 200 biophotonic imager as described in Materials and Methods. Tumor weight **C.** and Ki67 positivity **D.** were also described in Materials and Methods. Values are means ± standard errors. * *P* < 0.05, ** *P* < 0.01 *vs*. Veh. Veh: vehicle.

Next, we investigated the antitumor potential of ZER *in vivo via* oral administration in an orthotopic xenografts model using MDA-MB231-Luc cells. We injected MDA-231-Luc cells (5 × 10^6^ cell/100 μl) into the right secondary mammary fat. In the orthotopic model, tumor volume was suppressed by ZER treatment compared with vehicle-treated mice (Figure 7A). In addition, mice treated with 20 mg/kg ZER (4.7 ± 2.8 × 10^9^ photons/sec) developed significantly smaller tumors than the control mice treated with vehicle (70.4 ± 3.1 × 10^9^ photons/sec) as measured by *in vivo* luciferase activity (Figure 7B). We checked that the tumor weight level of ZER treated mice (1.22 ± 0.35 g) also decreased in compared with vehicle treated mice (2.56 ± 0.68 g) (Figure 7C). Finally, we further evaluated the effect of ZER on the expression of Ki-67 which is a marker of proliferation. As shown in Figure 7D, the expression of Ki-67 was downregulated in ZER treated mice as compared with vehicle treated mice. The positivity of Ki-67 decreased to 37.5 ± 5.1% of the vehicle level at 20 mg/kg ZER treatment (Figure 7D). In addition, we observed that FN expression was significantly decreased by ZER treated mice, although TGF-β1 expression did not change (Supplement Figure 2). Finally, we investigated the effect of ZER on metastasis using the 4T1 mammary carcinoma cells which are highly tumorigenic and invasive model. As shown in Supplement Figure 3A, we observed that ZER completely suppressed the metastatic potential of 4T1 cells xenograft tumors. Vehicle treated mice manifested large metastatic nodules in the lung, whereas these metastatic nodules significant decreased in ZER treated mice (Supplement Figure 3A). Especially, the number of metastatic nodules of ZER treated mice (9.6 ± 2.7 nodules) also decreased in compared with vehicle treated mice (27.1 ± 4.9 nodules) (Supplement Figure 3B). Based on these results, we demonstrated that ZER significantly suppresses the growth and metastatic potential of TNBC xenograft tumors.

## DISCUSSION

TGF-βs can modulate a variety of biological events such as cell growth, extracellular matrix formation, angiogenesis, and cancer progression [31, 32]. Plasma levels of TGF-βs are elevated in patients with breast cancer whereas after surgical removal of the primary tumor, highly expressed TGF-β1 levels normalize in the majority of patients [33, 34]. In addition, elevated TGF-β1 is correlated with increased tumor invasiveness and disease progression in a variety of tumors such as breast cancer and colorectal cancer [34, 35]. TGF-β-responsive gene signature is highly activated in chemotherapy-treated triple negative breast cancer (TNBC) when compared to other subtypes [29]. Consistent with these reports, we found that the level of TGF-β1 expression and invasiveness is higher in TNBC cells than in non-TNBC cells. On the other hand, the motility of TNBC cells, including invasion and migration is significantly decreased by LY2109761, a novel selective TGF-β receptor type I/II (TβRI/II) dual inhibitor. Therefore, we demonstrate that aberrant TGF-β1 expression directly or indirectly regulates the aggressive and invasive properties of TNBC cells.

TGF-β1 is also regarded as a metastasis inducer and promotes EMT associated the induction of various genes such as FN and MMPs with increased tumor cell motility [36, 37]. Increased FN expression level is associated with tumor progression and poor prognoses in breast cancer patients [38]. FN expression is closely involved with the growth and/or metastasis of surrounding tumor cells through the induction of EMT [39, 40]. We have also reported that FN treatment significantly augmented the rates of invasion and adhesion while FN-induced cell invasion was suppressed by the FN inhibitor (RGD tetrapeptide) in breast cancer cells [41]. Consistent with these reports, we also found that basal level of FN is augmented in TNBC cells. In contrast, highly expressed FN level is decreased TGF-β receptor type I/II dual inhibitor in a dose-dependent manner in Hs578T cells. Therefore, we demonstrate that blocking of TGF-β1 signaling pathway by TGF-β receptor type I/II dual inhibitors mediates the downregualtion of FN expression and then may directly or indirectly affect the motility of TNBC cells.

Although we could not fine the relationship between FN and MMPs expression in recent study [41], secreted FN enhances MMP-2 and MMP-9 expression through the MAPK and the PI3-K/Akt-dependent pathways in breast and ovarian cancer cells [19, 42]. Elevated expression of MMP-2 or MMP-9 is associated with human cancer invasion and metastasis [20]. In this study, we observed that the invasiveness of TNBC cells is significantly increased when compared to that of non-TNBC cells. Especially, the level of MMP-2 mRNA expression is higher in TNBC cells than in non-TNBC cells. On the other hand, elevated MMP-2 expression was decreased by TGF-β receptor type I/II dual inhibitors. Therefore, we suggest that MMP-2 expression is also regulated through the TGF-β dependent signalling pathway. In addition, increased MMP-2 as well as TGF-β1-induced MMP-2 and MMP-9 expression is associated with TNBC progression and metastasis.

TGF-β1 can stimulate the motility of many cancer cells such as prostate and breast cancer through in a variety of signaling pathway [15, 28, 34]. The activation of mitogen-activated protein kinase (MAPK) such as the extracellular signal-regulated kinase 1/2 (ERK1/2) and the p38 MAP kinase (p38 MAPK) in response to TGF-β1 triggers cellular migration and invasion [43]. Consistent with these reports, our results showed that TGF-β1 induces the activity of ERK and smad3 in HCC1806 TNBC cells. Interestingly, TGF-β1-stimulated FN and MMP-9 expression is suppressed by UO126 but not MMP-2. However, the induction of FN, MMP-9 and MMP-2 by TGF-β1 is suppressed by SIS3 treatment. Therefore, we demonstrate that TGF-1/smad3 signaling pathway is regarded as a metastatic modulator on the induction of FN, MMP-9, and MMP-2 by TGF-β1 in TNBC cells.

Zerumbone (ZER), a sesquiterpene in subtropical ginger, has been reported to inhibit tumor growth and to induce apoptosis against a wide variety of tumor cells, including colon cancer and leukemia cells [23, 30]. Murakami et al. reported that ZER suppresses 7,12-dimethylbenz[α]anthracene- and TPA-induced initiation and promotion of skin tumors [44]. Consistent with these reports, we observed that the tumorigenecity of MDA-MB231 cells and the Ki67 positivity were also decreased by ZER treatment. In addition, ZER inhibits the de novo synthesis of iNOS and COX-2 through the suppression of NF-κB and Iκ-Bα kinase activation [21]. Recently, we reported that ZER abolishes CD44 expression through the inhibition of EGF/STAT-3 pathway in breast cancer cells [45]. Furthermore, ZER significantly decreased cell invasion of TNBC through the suppression of IL-8 and MMP-3 expression [24]. In this study, we found for the first time that ZER significantly suppresses TGF-β1-induced FN, MMP-2, and MMP-9 expression through the inhibition of smad3 phosphorylation. Therefore, we demonstrate that ZER acts as a novel drug for suppression of cell motility and tumor growth of TNBC cells.

Here, we investigated the effect of ZER as a therapeutic drug for treatment of TNBC. We found for the first time that the levels of TGF-β1 mRNA expression significantly increased in TNBC cells compared with other subtypes. In addition, TNBC cells highly expressed the invasion related genes, including FN, MMP-2, and MMP-9 as well as the invasion rates is also significantly increased in TNBC cells compared with other subtypes. As expected, TGF-β receptor I/II inhibitor, LY2109761 significantly decreased not only the invasion and migration of TNBC cells but also the level of FN and MMP-2 expression. Therefore, our data establish that aberrant TGF-β1 expression influences the invasion and migration of TNBC cells through the induction of FN, MMP-2, and MMP-9. Interestingly, ZER dose-dependently suppressed the levels of basal- and TGF-β1-induced FN, MMP-2, and MMP-9 expression in HCC1806 TNBC cells through the inhibition of smad3 activity. In addition, ZER effectively suppresses the growth and metastatic potential of TNBC xenograft tumors. So, we suggest that ZER can be considered a promising targetable drug for TNBC treatment.

## MATERIALS AND METHODS

### Reagents

Dulbecco's modified Eagle's medium (DMEM), RPMI1640 and the antibiotics were purchased from Life Technologies (Rockville, MD, USA). Fetal bovine serum (FBS) was purchased from Hyclone (Logan, UT, USA). Twenty four-well invasion chambers (8 μm pore) were obtained from Becton-Dickinson (San Diego, CA). ZER was a generous gift from Dr. Murakami Akira (Kyoto University, Japan). LY2109761 was purchased from Selleck Chemicals (Houston, TX, USA). UO126 (a MEK1/2 inhibitor) were purchased from Tocris (Ellisville, MO, USA). SIS3 (a smad3 inhibitor) was purchased from Calbiochem (San Diego, CA, USA). The secondary horseradish peroxidase (HRP)-conjugated and mouse monoclonal anti-β-actin antibodies were purchased from Santa Cruz Biotechnology, Inc. (Santa Cruz, CA, USA). Mouse monoclonal anti-human Ki-67 antigen was purchased from Dako (Cambridgeshire, UK). Rabbit monoclonal anti-FN and smad3 (total and phospho-form) antibodies were purchased from Epitomics (Burlingame, CA, USA). Rabbit polyclonal ERK1/2 (total and phospho-form) antibodies were purchased from Cell Signaling Technology (Beverly, MA, USA). Recombinant Human TGF-β1 was purchased from R&D Systems (Minneapolis, MN, USA). The ECL prime reagents were from Amersham (Buckinghamshire, UK).

### Cell culture

MDA-MB231, and Hs578T human breast cancer cells were grown in a humidified atmosphere of 95% air and 5% CO_2_ at 37°C in DMEM supplemented with 10% FBS, 2 mM glutamine, 100 IU/ml penicillin and 100 μg/ml streptomycin. BT474, ZR75-1, SKBR3, and HCC1806 human breast cancer cells were grown in RPMI1640 media under the same conditions. Each cell line was maintained in culture medium supplemented without FBS for 24 h.

### Treatment with ZER and specific inhibitors

Cells were maintained in culture medium without FBS for 24 h, and then the culture media was replaced with fresh media without FBS. Breast cancer cells were pretreated with 10 or 20 μM concentrations of ZER for 16 h prior to the TGF-β1 treatment and then treated with 10 ng/ml TGF-β1 for 24 h. Furthermore, this experiments involving specific inhibitors such as UO126 and SIS3, was pretreated with specific inhibitors for 30 min prior to treatment with TGF-β1 and then they were treated with TGF-β1 for 24 h.

### Cell proliferation assay

The cell viabilities were analyzed by Quick Cell Proliferation Assay Kit II (BioVision, Mountain View, CA) according to the manufacturer's instructions. Briefly, HCC1806 (TNBC type) breast cancer cells (5×10^4^/well) were cultured in a 96-well plate in 100 μl/well of culture media in the absence or presence of the indicated dose of ZER. After incubating the cells for 24 h, 10 μl WST reagent was added to each well. Viable cells were quantified photometrically at 450 nm.

### Cell cycle analysis

Trypsinized cell pellets were resuspended in 1ml PBS and fixed in 70% ethanol for 20 min at room temperature (RT). The fixed cells were centrifuged at 1,500 rpm for 5 min and washed twice in PBS. The cells were resuspended in 1ml PBS with 100 μg/ml DNase-free RNase A (Biopure, Canada) and then incubated for 30 min in a 37°C water bath. The cells were then collected by centrifugation at 1,500 rpm and the cell pellets were washed twice with PBS. The cell pellets were then resuspended in PBS containing 50 μg/ml of propidium iodide (Sigma, St. Louis, MO, USA) and analyzed using the FACS-vantage (Becton-Dickinson, San Diego, CA, USA).

### Real-time PCR

The total RNA was extracted from the cells by using the TRIzol reagent (Invitrogen, Carlsbad, CA), according to the manufacturer's instructions. Isolated RNA samples were then used for RT-PCR. Samples (1 μg of total RNA) were reverse-transcribed into cDNA in 20 μl reaction volumes using a first-strand cDNA synthesis kit for RT-PCR, according to the manufacturer's instructions (MBI Fermentas, Hanover, MD, USA).

The gene expression was quantified by Real-Time PCR using a SensiMix SYBR Kit (Bioline Ltd., London, UK) and 100 ng of cDNA per reaction. The specific primer sets used to detection of each gene mRNA expression (Table 1). An annealing temperature of 60°C was used for all of the primers. PCRs were performed in a standard 384-well plate format with an ABI 7900HT real-time PCR detection system. For data analysis, the raw threshold cycle (C*_T_*) value was first normalized to the housekeeping gene for each sample to get the ΔC*_T_*. The normalized ΔC*_T_* was then calibrated to the control cell samples to get the ΔΔC*_T_*.

**Table 1:** Specific primer sequences for analysis of TGF-β1, FN, MMP-2, MMP-9, and GAPDH mRNA expression

Gene name	Forward	Reverse
TGF-β1	TGA ACC GGC CTT TCC TGC TTC TCA TG	GCG GAA GTC AAT GTA CAG CTG CCG C
FN	CCA CCC CCA TAA GGC ATA GG	GTA GGG GTC AAA GCA CGA GTC ATC
MMP-2	GGC CTC GTA TAC CGC ATC AAT C	GGC CTC TCC TGA CAT TGA CCT T
MMP-9	CCC GGA CCA AGG ATA CAG	GGC TTT CTC TCG GTA CTG
GAPDH	ATT GTT GCC ATC AAT GAC CC	AGT AGA GGC AGG GAT GAT GT

### ELISA

ELISA assays were performed on culture media (400 μL) collected from BT474 and MDA231 breast cancer cells. Protein levels of TGF-β1 was measured using an ELISA kit for human TGF-β1 (Koma Biotech, Seoul, Korea), according to the manufacturer's instructions and then using a microtiter plate reader, read the plate at 450 nm wavelength.

### Wound healing assay

MDA231, Hs578T, and HCC1806 TNBC cells were seeded in 6-well plates and cultured for 24 h. Then, the TNBC cells were maintained in culture medium without FBS for 16-24 h. The cell monolayer was scratched with a 200-μL pipette tip to create a wound, which was washed twice with PBS to remove the suspended cells. TNBC cells were maintained with or without 10 μM LY2109761 for 24 h in serum-containing medium. The cells migrating from the leading edge were photographed at 0 and 24 h using a CK40 inverted microscope (Olympus, Tokyo, Japan). Cell to cell distance was analyzed average of pixel number on three sites using Axiovision software (Carlzeiss, Thornwood, NY).

### Cell invasion assay

Matrigel-coated filter inserts (8 μm pore size) that fit into 24-well invasion chambers were obtained from Becton-Dickinson. Breast cancer cells to be tested for invasion were resuspended in culture medium (5 × 10^5^ cells/well) and then added to the matrigel-coated upper compartment of the invasion chamber in the presence or absence of 20 μM ZER. Fresh culture media with 5% FBS were added to the lower compartment of the invasion chamber. The chambers were incubated at 37°C for 24 h. After incubation, the cells on the upper side of the filter were removed using cotton swabs, and the bottom filters were fixed in 100% methanol, washed in 1× PBS, and stained using toluidine blue dye. Breast cancer cells that invaded through the matrigel were located on the underside of the filter. These cells were photographed using a Scanscope XT apparatus (Aperio Technologies Inc., CA, USA). The invasion rates were calculated by averaging the total number of cells from four filters.

### Western blotting

The cell culture media (supernatants) and cell lysates were used in the immunoblot analysis for FN, ERK, smad3, and β-actin. The proteins were boiled for 5 min in Laemmli sample buffer and then they were electrophoresed in 8% or 10% SDS-PAGE gels, respectively. The separated proteins were transferred to PVDF membranes and the membranes were then blocked with 10% skim milk in TBS with 0.01% Tween-20 for 15 min. The blots were incubated with anti-FN, ERK (total and phospho-form), Akt (total and phospho-form), and β-actin antibodies (1/1,000 dilution) in 1% TBS/T buffer (0.01% Tween 20 in TBS) at 4°C overnight. The blots were washed 3 times in TBS with 0.01% Tween 20 and they were subsequently incubated with anti-rabbit peroxidase-conjugated antibody (1/2,000 dilution) in TBS/T buffer. After 1 h incubation at room temperature (RT), the blots were washed three times and ECL prime reagents were used for development.

### Zymography

Zymography was performed in 10% polyacrylamide gels that had been cast in the presence of gelatin, as described previously [42]. Briefly, samples (100 μl) were resuspended in loading buffer, and run on a 10% SDS-PAGE gel containing 0.5 mg/ml gelatin without prior denaturation. After electrophoresis, the gels were washed to remove SDS and incubated for 30 min at room temperature (RT) in a renaturing buffer (50 mM Tris, 5 mM CaCl_2_, 0.02% NaN_3_, and 1% Triton X-100). Next, the gels were incubated for 48 h at 37°C in a developing buffer (50 mM Tris-HCl [pH 7.<8], 5 mM CaCl_2_, 0.15 M NaCl, and 1% Triton X-100). The gels were subsequently stained with Coomassie brilliant blue G-250, destained in 30% methanol, and flooded with 10% acetic acid to detect gelatinase secretion.

### Adenovirus transfer

The empty (*Lac Z*) and adenoviral human FLAG-tagged smad3 cDNA were gifts of Dr. Seok Hee Park (Sungkyunkwan University, Seoul, Korea). Recombinant adenovirus expressing human smad3 was reproduced into 293A cells. The expression of this construct was confirmed by western blotting.

### The measurement of tumorigenecity in an orthotopic xenograft model

In order to establish a nude mice xenograft model, we used 6- to 8-week-old female Balb/c nude mice (Orient Bio, Korea), weighing about 18-22 g. Mice were kept in pathogen-free animal housing in accordance with the National Institutes of Health Guide for the Care and Use of Laboratory Animals.

The firefly luciferase 2 (Luc2) and td Tomato (L2T) fusion constructs were gifts of Dr. Sanjiv Sam Gambhir (Stanford University, CA, USA) [46]. Stable MDA-MB231-Luc cells were cultured and resuspended in matrigel (BD Biosciences, Bedford, MA, USA) to give a final concentration of 5 × 10^6^ cells/100 μL, which was injected directly into the right secondary mammary fat. The mice were randomly divided into two groups (*n* = 5/group), treated with vehicle (1 × PBS) or ZER (20 mg/kg body weight in vehicle) by oral injection three times a week for 28 days. The tumor size of the vehicle and the ZER-treated mice was measured using digital calipers at the set time point and the volume was measured using the formula: V = 1/2 length × (width)^2^. Growth curves were calculated using average relative tumor volume within each group (vehicle or ZER-treated group) at the set time points. Bioluminescent imaging analysis was done by a Xenogen IVIS^®^ Spectrum (PerkinElmer). Mice were injected intraperitoneally with 100 μl of D-luciferin (10 mg/ml) in PBS, and after 10 min, imaged under anesthesia with 2.5% isofluorane. Luminescence images were captured as photons/sec/ROI (region of interest) minus background luminescence for a similarly sized region.

### Immunohistochemical staining of MDA-MB231-Luc cells xenograft tumor samples

MDA-MB231-Luc xenograft tumors harvested from vehicle and ZER treated mice were fixed with formalin and embedded in paraffin. Tissue sections were cut and deparafinized in xylene, and dehydrated in graded alcohol and hydrated in water. Tissue sections (4 μm) were subjected to immunohistochemical staining using Ki67 rabbit polyclonal (Dako, Glostrup, Denmark), FN rabbit monoclonal (Abcam, Cambridge, UK) and TGF-β1 rabbit polyclonal antibodies (Santa Cruz, CA, USA) by a Bond-Max automated staining system (Leica). The Ki67 positivity of cells was analyzed using a Scanscope XT from Aperio (Vista, CA).

### Statistical analysis

Statistical significance was determined using Student's *t*-test. Data are presented as means ± SEM. All quoted *P* values are two-tailed and differences were considered significant for *P values* < 0.05. Microsoft Excel was used for the statistical analyses.

## SUPPLEMENTARY MATERIAL METHODS AND FIGURES


