# Supplementary material for: Zerumbone suppresses the motility and tumorigenecity of triple negative breast cancer cells via the inhibition of TGF-β1 signaling pathway
Source: Oncotarget. 2015 Nov 30;7(2):1544–58. doi: 10.18632/oncotarget.6441 (PMC4811479; doi:10.18632/oncotarget.6441)
Supplement: Supplementary file 1 [file oncotarget-07-1544-s001.pdf]

Zerumbone suppresses the motility and tumorigenecity of triple negative breast cancer cells via the inhibition of TGF-β1 signaling pathway

Supplementary Material

Supplement methods

TGF-β receptor I and TGF-β receptor II siRNA transfection

TGF-β receptor I (TGFBRI) and TGF-β receptor II (TGFBRII) siRNA were purchased from Bioneer Corporation (Daejeon, Korea). A synthetic siRNA against TGF-β receptor I and TGF-β receptor II mRNA was used to inhibit endogenous TGFBRI and TGFBRII gene expression. The duplex sequences of TGFBRI and TGFBRII siRNA used for this experiment are as follows: human TGFBRI siRNA [sense-GAA CAA UUG CGA GAA CUA U (dTdT); antisense-AUA GUU CUC GCA AUU GUU C (dTdT)] and human TGFBRII siRNA [sense-GAG UUG CCA UAU CUG UCA UCA U(dTdT); antisense-AUG ACA GAU AUG GCA ACU C(dTdT)], We found that the optimal siRNA knock-down conditions involved transfecting HCC1806 and MDA231 breast cancer cells at 80% confluence maintained in RPMI or DMEM with 10% FBS, respectively. Effectene (Qiagen, Valencia, CA) was used for transfections with siRNA following protocols provided by the manufacturer. After the 72 h transfection, the levels of TGFBRI, TGFBRII, FN, MMP-2, and MMP-9 mRNA expression were analyzed by real-time PCR.

The measurement of metastatic potential in 4T1 cells orthotopic xenograft model

In order to establish a nude mice xenograft model, we used 6- to 8-week-old female Balb/c nude mice (Orient Bio, Korea), weighing about 18-22 g. Mice were kept in pathogen-free animal housing in accordance with the National Institutes of Health Guide for the Care and Use of Laboratory Animals.

4T1 mammary carcinoma cells (1 × 10^5 cells/100 μL) were injected directly into the right secondary mammary fat pad. The mice were randomly divided into two groups (n = 5/group), treated with vehicle (1 × PBS) or ZER (20 mg/kg body weight in vehicle) by oral injection three times a week for 24 days. Lung tissues harvested from vehicle and ZER treated mice were fixed with formalin and embedded in paraffin. Tissue sections were cut and deparafinized in xylene, and dehydrated in graded alcohol and hydrated in water. Tissue sections (4 μm) were subjected to immunohistochemical staining using hematoxylin and eosin (H & E). The slides were analyzed using the Scanscope XT from Aperio (Vista, CA).

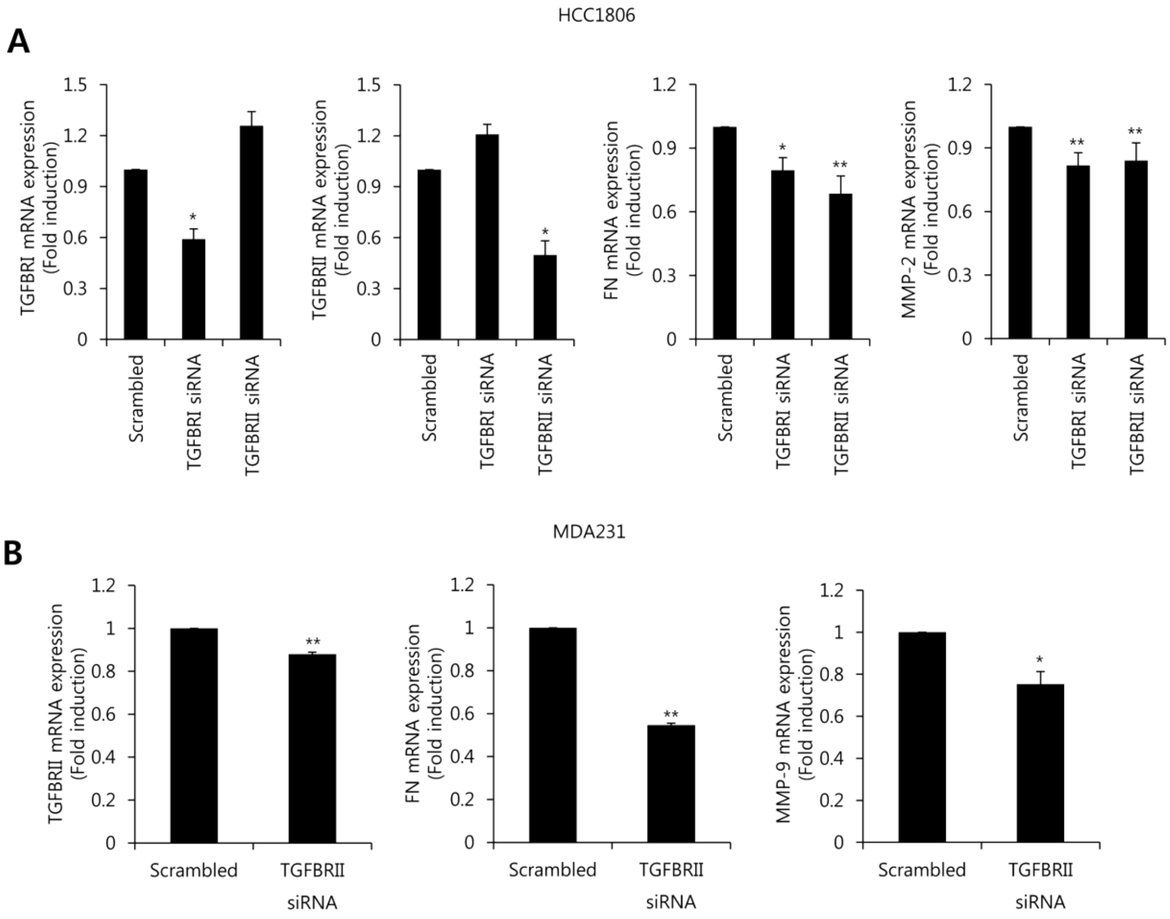

Supplement Figure 1: The basal levels of FN, MMP-2, and MMP-9 expression are decreased by TGF-β receptor I or TGF-β receptor II siRNA overexpression in TNBC cells. (A) HCC1806 cells were transfected with TGF-β receptor I (TGFBRI) or TGF-β receptor II (TGFBRII) siRNA, respectively, for 72 h. The levels of TGFBRI, TGFBRII, FN, and MMP-2 mRNA expression were analyzed by real-time PCR. (B) MDA231 cells were transfected with TGFBRII siRNA for 72 h. The levels of TGFBRII, FN, and MMP-9 mRNA expression were analyzed by real-time PCR. The results are representative of three independent experiments. Values are means ± standard errors. *$P < 0.05$, **$P < 0.01$ *vs.* Scrambled.

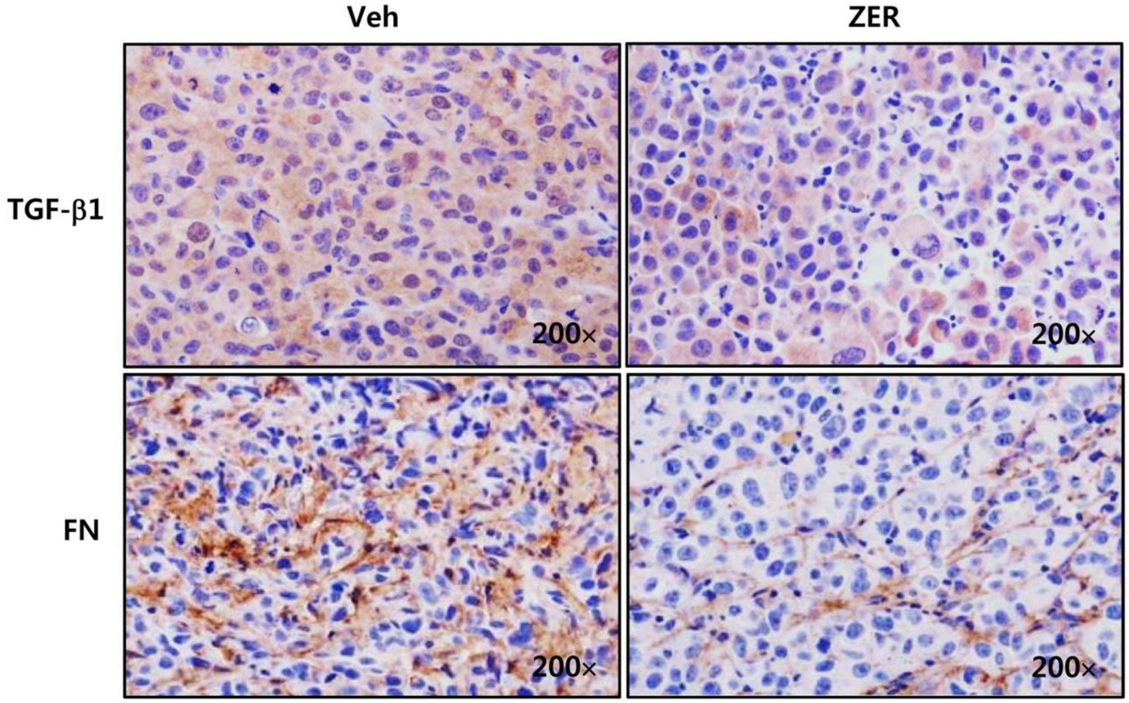

Supplement Figure 2: ZER suppresses the level of FN expression in MDA231 xenograft tumors. Mice were injected with MDA231 TNBC cells. After 28 days, tumor tissues of MDA231 xenograft were subjected to immunohistochemical staining using TGF-β1 and FN antibodies as described in Materials and Methods. The slides were analyzed using the Scanscope XT.

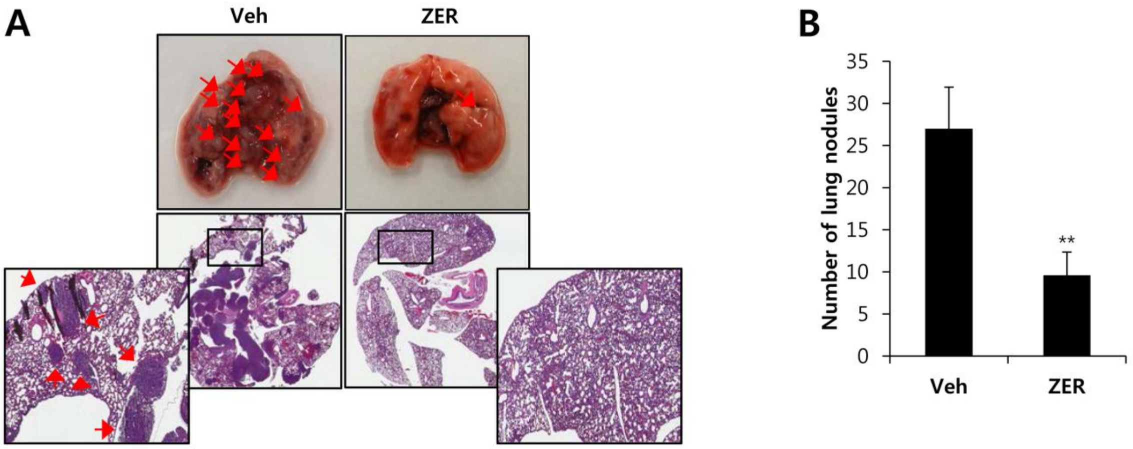

Supplement Figure 3: ZER suppresses metastatic potential of 4T1 cells orthotopic xenograft model. (A) Secondary mammary fat pad of mice was injected with 4T1 TNBC cells. After 24 days, we removed lung tissues for Veh- and ZER-treated groups (n=5) and analyzed metastatic events. Red arrows indicated metastatic nodules. (B) The number of metastatic nodules was counted in lung tissues. Values are means ± standard errors. $^{**}P < 0.01$ *vs.* Veh. Veh: vehicle, ZER: zerumbone.
